# Survival and Biodistribution of Xenogenic Adipose Mesenchymal Stem Cells Is Not Affected by the Degree of Inflammation in Arthritis

**DOI:** 10.1371/journal.pone.0114962

**Published:** 2015-01-05

**Authors:** Karine Toupet, Marie Maumus, Patricia Luz-Crawford, Eleuterio Lombardo, Juan Lopez-Belmonte, Peter van Lent, Marina I. Garin, Wim van den Berg, Wilfried Dalemans, Christian Jorgensen, Danièle Noël

**Affiliations:** 1 U 844, Inserm, Hôpital Saint-Eloi, Montpellier, France; 2 UFR de Médecine, Université Montpellier1, Montpellier, France; 3 TiGenix, Madrid, Spain; 4 Farma-cros Iberica, Albacete, Spain; 5 Experimental Rheumatology, Radboud University Medical Center, Nijmegen, Netherlands; 6 CIEMAT/CIBERER, Madrid, Spain; 7 TiGenix, Leuven, Belgium; 8 Service d’Immuno-Rhumatologie Thérapeutique, Hôpital Lapeyronie, Montpellier, France; Georgia Regents University, UNITED STATES

## Abstract

**Background:**

Application of mesenchymal stem/stromal cells (MSCs) in treating different disorders, in particular osteo-articular diseases, is currently under investigation. We have already documented the safety of administrating human adipose tissue-derived stromal MSCs (hASCs) in immunodeficient mice. In the present study, we investigated whether the persistence of MSC is affected by the degree of inflammation and related to the therapeutic effect in two inflammatory models of arthritis.

**Methodology/Principal Findings:**

We used C57BL/6 or DBA/1 mice to develop collagenase-induced osteoarthritis (CIOA) or collagen-induced arthritis (CIA), respectively. Normal and diseased mice were administered 2.5×10^5^ hASCs in the knee joints (IA) or 10^6^ in the tail vein (IV). For CIA, clinical scores were monitored during the time course of the disease while for CIOA, OA scores were assessed by histology at euthanasia. Thirteen tissues were recovered at different time points and processed for real-time PCR and *Alu* sequence detection. Immunological analyses were performed at euthanasia. After IV infusion, no significant difference in the percentage of hASCs was quantified in the lungs of normal and CIA mice at day 1 while no cell was detected at day 10 taking into account the sensitivity of the assay, indicating that a high level of inflammation did not affect the persistence of cells. In CIOA mice, we reported the therapeutic efficacy of hASCs at reducing OA clinical scores at day 42 when hASCs were not detected in the joints. However, the percentage and distribution of hASCs were similar in osteoarthritic and normal mice at day 1 and 10 after implantation indicating that moderate inflammation does not alter hASC persistence *in vivo*.

**Conclusions/Significance:**

While inflammatory signals are required for the immunosuppressive function of MSCs, they do not enhance their capacity to survive *in vivo*, as evaluated in two xenogeneic inflammatory pre-clinical models of arthritis.

## Introduction

Mesenchymal stem or stromal cells (MSCs) are promising cells for regenerative medicine, based on their differentiation, trophic or immunomodulatory properties [1]. MSCs originating from bone marrow (BM-MSCs) or fat tissue (ASCs) are the most widely used in clinical trials for a wide range of diseases. Depending on the diseases, MSCs may be systemically or locally injected. For osteo-articular diseases, intravenous (IV) injection will be favored in case of systemic diseases such as rheumatoid arthritis (RA) which is characterized by the concurrent inflammation of several joints [2, 3]. In the case of osteoarthritis (OA), where only one or two joints are affected, intra-articular (IA) injection of cells could likely be the most relevant option. Besides the importance of the route of administration, priming of MSC by inflammatory signals is proposed to be required for therapeutic efficacy, in particular for stimulating their immunosuppressive effects [4].

An inflammatory pathological environment, as present in patients with dysregulated immune functions, is known to induce MSC immunosuppressive functions, as recapitulated *in vitro* using MSC induction with interferon (IFN)-γ and tumor necrosis factor (TNF)-α or interleukin (IL)-1α or -1β [5]. A role of inflammatory signals on MSC activation was also suggested in one of our previous studies reporting that in vitro coculture of hASCs with highly inflammatory chondrocytes from OA patients down-regulated the expression of several inflammatory molecules while no effect was observed when chondrocytes expressed low or moderate levels of inflammatory molecules [6]. This is further supported by recent findings that the protective effect of hASCs was only observed in the collagenase-induced model of osteoarthritis when high synovial inflammation was present at the time of injection[7]. In animal models of transplantation, MSCs were effective in prolonging graft survival when injected before transplantation while they were not when injected later on [8]. These observed differences may be due to distinct suppressive responses of MSCs in the absence or presence of inflammatory mediators. They may also be related to a different survival rate of MSCs submitted to inflammatory stimuli.

We previously showed different survival time and biodistribution depending on the route of administration using autologous human ASCs (hASCs) implantation in normal SCID/Bg mice, which do not mount inflammatory response to foreign cells [9]. However, such studies in immunodeficient mice do not take into account the pathological environment of diseases. The role of the inflammatory milieu on the therapeutic efficacy of MSCs has been widely claimed but whether it may also be related to a better cell survival has been poorly investigated in vivo. The main objective was to investigate whether the degree of inflammation may influence the survival of MSCs since their capacity to escape the host immune response is proposed to be related to a priming/activation step by the inflammatory environment. In the present study, we therefore investigated the therapeutic efficacy and persistence of hASCs in mice in the context of moderate or severe inflammation in murine models of collagenase-induced osteoarthritis (CIOA) and collagen-induced inflammatory arthritis (CIA), respectively.

## Methods

### Isolation and characterization of adipose tissue-derived mesenchymal stem cells

Human samples were obtained from patients after informed consent as approved by the French Ministry of Research and Innovation (approval DC-2010-1185) or the Spanish Ethics Committee of reference (Clinica de la Luz Hospital) for the site of tissue procurement. hASCs were isolated from normal donors undergoing plastic surgery after informed written consent as described [10, 11]. Briefly, fat tissue was digested with collagenase at 37°C for 1h. The stromal vascular fraction was plated in α-MEM medium supplemented with 100U/ml penicillin/streptomycin, 2mmol/mL glutamine and 10% fetal bovine serum. hASCs were characterized by their immunophenotype (positive for CD13, CD73, CD90, CD105 and negative for CD11b, CD14, CD31, CD34, CD45) and their trilineage potential of differentiation as published [10]. Cells were used before 14 population doublings.

### CIOA animal model

Ten weeks old C57BL/6 male mice were obtained from Janvier (Saint-Berthevin). Animal experimentation was conducted in agreement with the Languedoc-Roussillon Regional Ethics Committee on Animal Experimentation (approval CEEA-LR-10041). All surgery was performed under isoflurane gas anesthesia, and all efforts were made to minimize suffering. Knee joints were injected with 1 unit of type VII collagenase from *Clostridium histolyticum* (Sigma-Aldrich, L’Isle d’Abeau) in 5μl of saline on days 0 and 2, as previously described [12]. On day 7, two groups of 10 mice received either saline (CIOA) or hASCs (2.5×10^5^ cells/7μl; CIOA+ASC), which were administered in both knee joints. Animals from each group were sacrificed at day 42 for histological evaluation of the disease and blood recovery. In parallel, either normal or CIOA groups of 30 animals received 2.5×10^5^ cells hASCs/7μl on day 7. Euthanasia was performed on 10 animals at day 1, 10 or 30 for DNA analysis.

### CIA animal model

Animal experimentation was conducted in agreement with the Spanish Real Decreto 223/1988, the Directive 86/609/CEE and also Directive 2010/63/EU on the protection of animals used for scientific purposes for the curative approach. For the preventive approach, authorization was obtained from the Languedoc-Roussillon Regional Ethics Committee on Animal Experimentation (approval CEEA-LR-11052). All procedures were designed to minimize suffering. CIA was induced in male DBA/1 mice aged 7–9 weeks purchased from Janvier (Saint-Berthevin). Immunization and boost were performed by injecting 100μl of chicken collagen II in Freund’s complete and incomplete adjuvant (2 mg/ml) subcutaneously in the back respectively, at day 0 and 21. For the preventive approach, groups of 8 mice were infused IV with saline or hASCs (10^6^ cells/100μl saline) at day 18 and day 24. For the curative approach, saline or hASCs (10^6^ cells/100μl saline) were administered IV in groups of 10 mice when the arthritis score was 2–4 [13, 14]. In both approaches, arthritis score was determined by measurement of hind paw swelling and inflammatory reaction for hind and fore paws for 10 or 13 days after the last injection. Clinical score was assessed using the following system: grade 0, no swelling; grade 1: ≥0.1 mm increase of paw swelling or 1 digit; grade 2: ≥ 0.2 mm paw swelling increase and two groups of joints inflamed; grade 3: extensive swelling (≥ 0.3 mm) with severe joint deformity and more than two groups of joints inflamed; grade 4: pronounced swelling (≥ 0.45 mm) with pronounced joint deformity. A total score of 16 was recorded for each mouse. In parallel, 10^6^ hASCs were injected in one group of 30 mice scored 2–4 CIA or one group of 30 normal age-matched mice at the same time. Mice were euthanatized on day 1, 10, 30 for DNA analysis.

### Organ recovery

At sacrifice, 13 organs (lung, heart, kidney, spleen, *Tibialis anterior* muscle, brain, inguinal fat pad, stomach, intestine, liver, testis or ovaries, blood, total knee joint) were recovered and frozen at −80°C for DNA analysis, as described [9]. For the CIOA model, one hind paw was sectioned at the epiphyses of both femur and tibia to recover total knee joint exempt of muscle tissue for DNA analysis. The second hind paw was sectioned at half of the femur and at the ankle joint and fixed in 3.7% formaldehyde for histology.

### DNA extraction and real time PCR

All or part of the organs were mechanically dissociated and DNA extraction was performed using the DNeasy Blood & Tissue kit (Qiagen, Courtaboeuf) [9]. Real time PCR (qPCR) was performed on 25ng DNA in 10μl that contained 5μl of DNA Master SYBR Green I kit (Roche Diagnostics, Meylan) and 0.05μM primers. Sequences of primers and PCR conditions were as described [9]. Standard curves were generated by adding ten-fold serial dilutions of hASCs in murine MSCs (10^6^ total cell number). Results were expressed as the percentage of hASCs per organ or blood (total blood evaluated at 1.5ml/mouse) and limit of detection was estimated to be 0.005% hASCs of total cells [9].

### Cytokine measurement

Murine MCP-1 and S100A9 alarmin concentrations were determined in sera of CIOA mice at euthanasia by specific sandwich enzyme-linked immunosorbent assay (ELISA) as recommended by the supplier (R&D Systems).

### Histological analysis

Knee joints from CIOA mice were embedded in paraffin and cut in sections of 7μm, stained with Safranin O-Fast green for analysis of cartilage damage. Cartilage damage was scored using the modified Pritzker OARSI score [15]. In brief, lateral and medial tibia plateaus and femur condyles were scored for the grade of cartilage destruction (0–6) and the extent of damaged cartilage surface (0–5). For each location of a mouse knee, these scores were multiplied and an average of 3 scores corresponding to 3 sections separated by a 140μm thickness formed the OA cartilage score.

### T lymphocyte subset analysis by flow cytometry

Splenocytes and lymph node cells were isolated from CIA mice, as described previously [14]. For intracellular cytokine detection, cells were stimulated with PMA (50 ng/ml), ionomycin (1 μg/ml) and brefeldin A (10 μg/ml) for 4 h. Specific antibodies against mouse CD4 and CD25 were then added to the cells for 30 min before fixation and permeabilization with Perm/Fix solution (eBioscience, Paris). Cells were finally labelled with anti-IL-10, anti-IL-17, and anti-IFN-γ antibodies (BD Biosciences, Le Pont-de-Claix). Samples were analysed on a FACS Canto II and the analysis performed using the BD FACSdiva software (BD Biosciences).

### Statistical analysis

Statistical analysis was performed with GraphPad Software (San Diego, CA). Comparison between groups used the Shapiro-Wilk normality test. When groups passed normality test, an unpaired t test was performed; otherwise, a Mann-Whitney test was used.

## Results

### hASCs reduce the clinical score and the inflammatory response of arthritic mice

**Figure 1:**
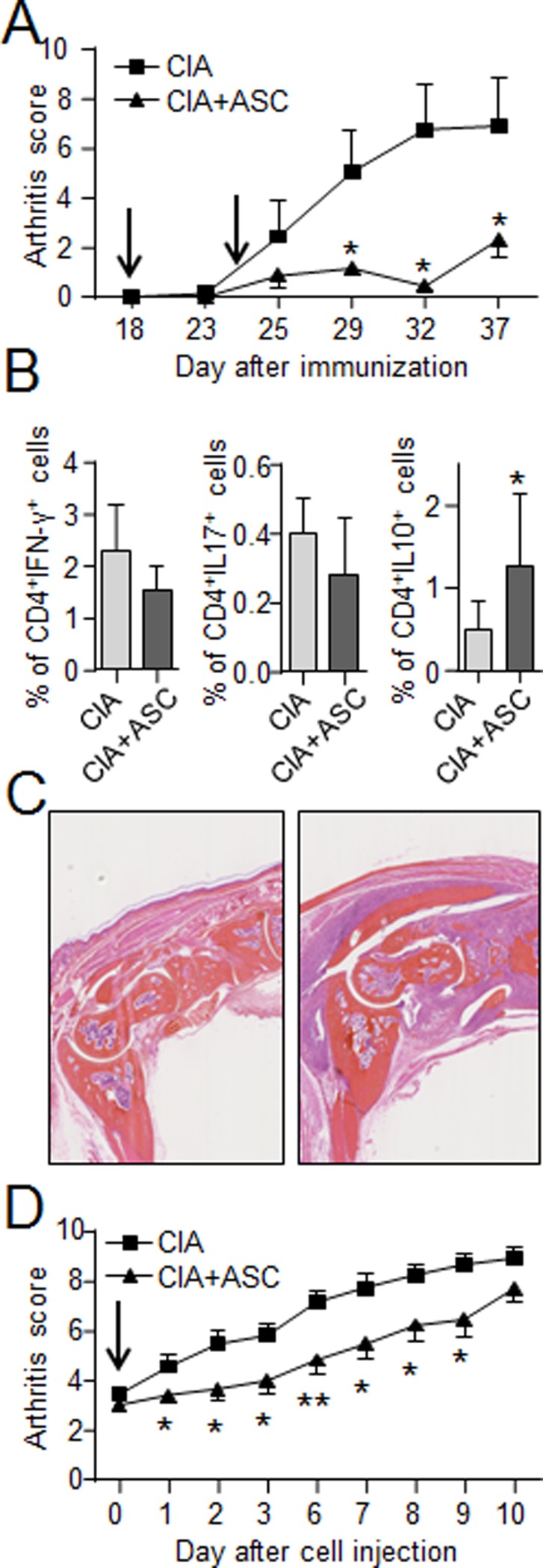
Effect of IV injection of hASCs in the CIA mouse model. (A) Arthritic score for hind and fore paw swelling at different time points during the course of the disease when hASCs were infused before disease onset (preventive approach). Arrows indicate the days of hASC injection. (B) Percentage of Th1 lymphocytes (CD4^+^IFN^+^), Th17 lymphocytes (CD4^+^IL17^+^) in spleens and Tr1 regulatory lymphocytes (CD4^+^IL10^+^) in lymph nodes of CIA and hASC-treated CIA mice. (C) Representative histological pictures of ankle joints from CIA (left) and hASC-treated CIA mice (right). (D) Arthritic score for hind and fore paw swelling at different time points during the course of the disease when hASCs were infused at disease onset (therapeutic approach). Results are expressed as the mean ± SEM (standard deviation of the mean), n = 8. *p<0.05, **p<0.01.

We first aimed at investigating the effect of hASCs in a model of severe and systemic inflammation in the collagen-induced arthritis (CIA) model. We evaluated two therapeutic strategies. The first one consisted of injecting hASCs at day 18 and day 24 just before the onset of CIA and at the very beginning of joint inflammation (prophylactic approach). In this model, hASCs efficiently reduced the arthritis symptoms for at least 20 days (Fig. 1A). Although the aim of the present study was not to determine precisely the mechanisms by which hASCs exerted their effect, we quantified the variations of the different T lymphocyte subsets in response to the treatment. We found a decrease of the number of CD4^+^IFN-γ^+^ Th1 cells and CD4^+^IL-17^+^ Th17 cells in the spleens of hASC-treated mice as compared to CIA mice (Fig. 1B). The same tendency was also observed in the lymph nodes of hASC-treated mice (data not shown). We also noticed a significant increase of CD4^+^IL-10^+^ Tr1-like lymphocytes in lymph nodes (Fig. 1B). The second strategy was to inject the hASCs after the onset of arthritis when mice reached an arthritic score between 2 and 4 (therapeutic approach). Using this approach, we also recorded a significant reduction of arthritic scores in hASC-treated mice for at least 10 days, although to a lesser extent than that observed when cells were injected before onset. In concordance with previous data using hASCs or hMSCs from amniotic membrane or umbilical cord [13, 16, 17], these data therefore confirmed that hASCs can exert their anti-inflammatory function in arthritic mice.

### Short-term persistence of hASCs after intravenous injection was not related to the inflammatory status of the mice

**Figure 2:**
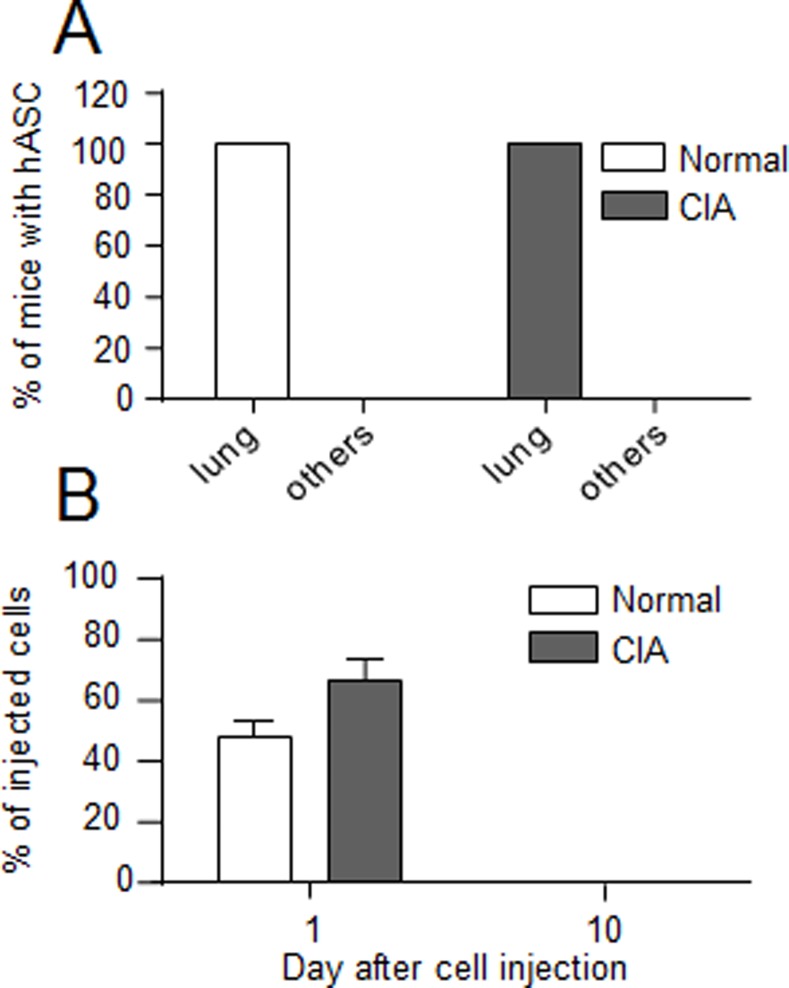
Comparison of the biodistribution of hASCs after IV administration in normal or CIA mice. (A) Percentage of mice in which hASCs were detected in the lungs or 12 other independent organs at day 1 after IV injection. (B) Percentage of hASCs detected in lung relative to the number of initially injected hASCs in normal or CIA mice. Results are expressed as the percentage of hASCs as evaluated by A*lu* sequence quantification and normalization using serial dilution of hASCs in murine MSCs ± SEM (standard deviation of the mean).

We determined the persistence and biodistribution of hASCs in arthritic mice that received the hASCs at disease onset, when the inflammatory response of the host is already very high and the therapeutic effect of hASCs more modest (therapeutic approach). At day 1 after IV administration in normal or CIA mice, hASCs were detected in the lungs in 10/10 injected mice (Fig. 2A). Cells were not detected in the other 12 organs. No significant difference in the percentage of hASCs present in the lungs was measured in normal (47.6 ± 5.9%) or arthritic (66.4 ± 6.9%) mice at that time point although some tendency to higher cell number in CIA mice was observed (Fig. 2B). At day 10, hASCs could not be detected in arthritic or normal animals. Although we could not evaluate the survival rate of cells between day 1 and 10, the data suggested that high levels of systemic inflammation did not affect the survival of hASCs as compared to normal conditions while their anti-inflammatory function contributed to reducing the arthritic symptoms.

### hASCs efficiently decrease the OA score after intra-articular injection in the CIOA mice

**Figure 3:**
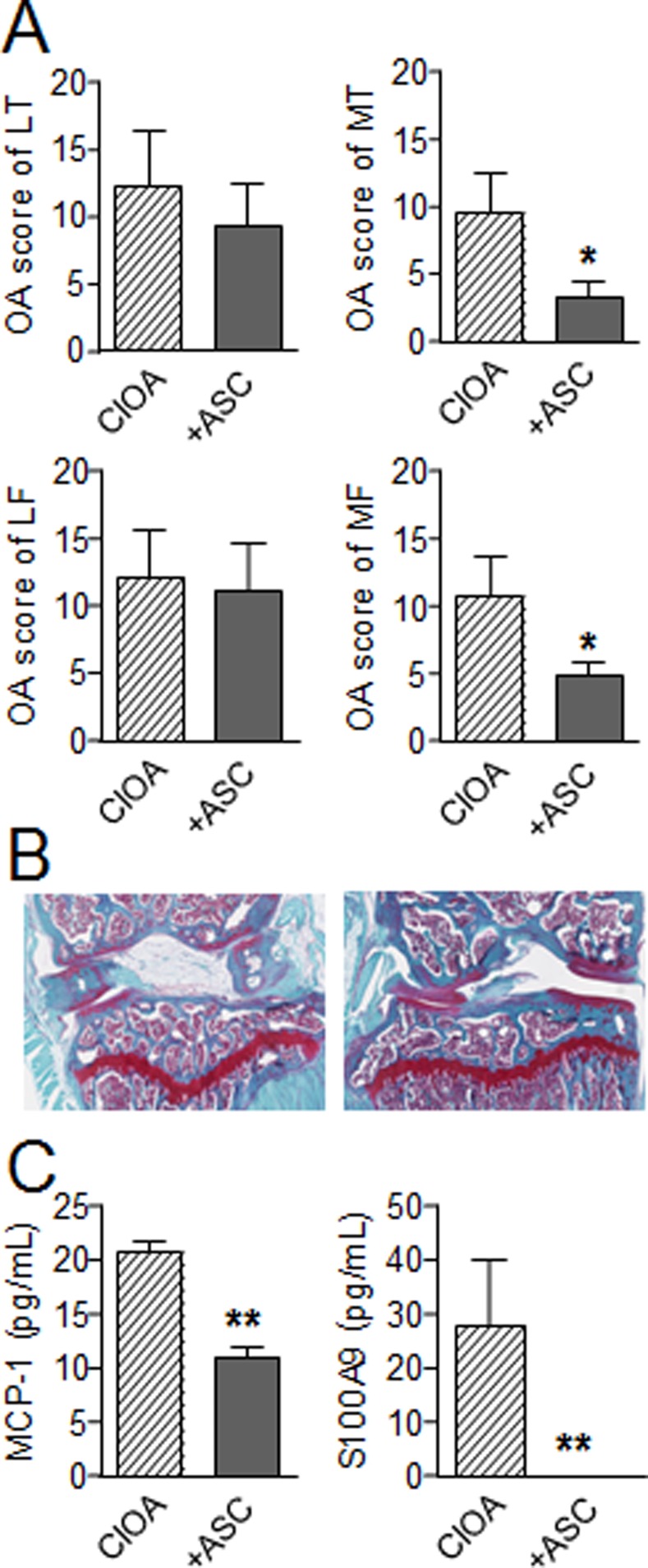
Effect of IA injection of hASCs in the CIOA mouse model. (A) OA score for **c**artilage destruction at 4 different locations in the knee joint, i.e., the lateral tibia plateau (LT), lateral femur condyle (LF), medial femur condyle (MF), and medial tibia plateau (MT) at euthanasia. (B) Representative photographs of knee joints from CIOA (left) and hASC-treated CIOA mice (right). (C) MCP-1 and S100A9 alarmin concentrations were determined in sera of mice at euthanasia by specific sandwich enzyme-linked immunosorbent assay (ELISA). Results are expressed as the mean ± SEM (standard deviation of the mean), n = 8. *p<0.05, **p<0.01.

We then aimed at evaluating the effect of hASCs in the CIOA model where a local moderate inflammation of the synovial membrane is reported. In this model, we previously described that the injection of murine ASCs significantly decreased the OA score [12]. We therefore determined whether hASCs may exert a similar effect after injection in the knee joints of mice at day 7 after CIOA induction. Histological analysis revealed a decrease of the OA scores in both lateral and medial compartments of the joints with significantly reduced scores in the median femur condyles and tibial plateaux 35 days after hASC implantation (Fig. 3A). Representative photographs of knee joints illustrated the high rate of degradation of articular cartilage in CIOA mice with depletion in safranin O-stained proteoglycans and cartilage erosion whereas protection of cartilage degradation was noticed in the ASC-treated mice (Fig. 3B). Concomitantly, a reduction of systemic inflammation as measured by the levels of alarmin S100A9 and MCP-1 in the sera of treated mice was observed at euthanasia (Fig. 3C). MIP-1A, RANKL and IL-1β were not detected in the sera of mice (data not shown). We therefore showed for the first time that hASCs are efficient at reducing the OA clinical scores in the CIOA model by decreasing synovial inflammation.

### hASCs administered by intra-articular injection were detected for at least 10 days in osteo-arthritic knee joints

**Figure 4:**
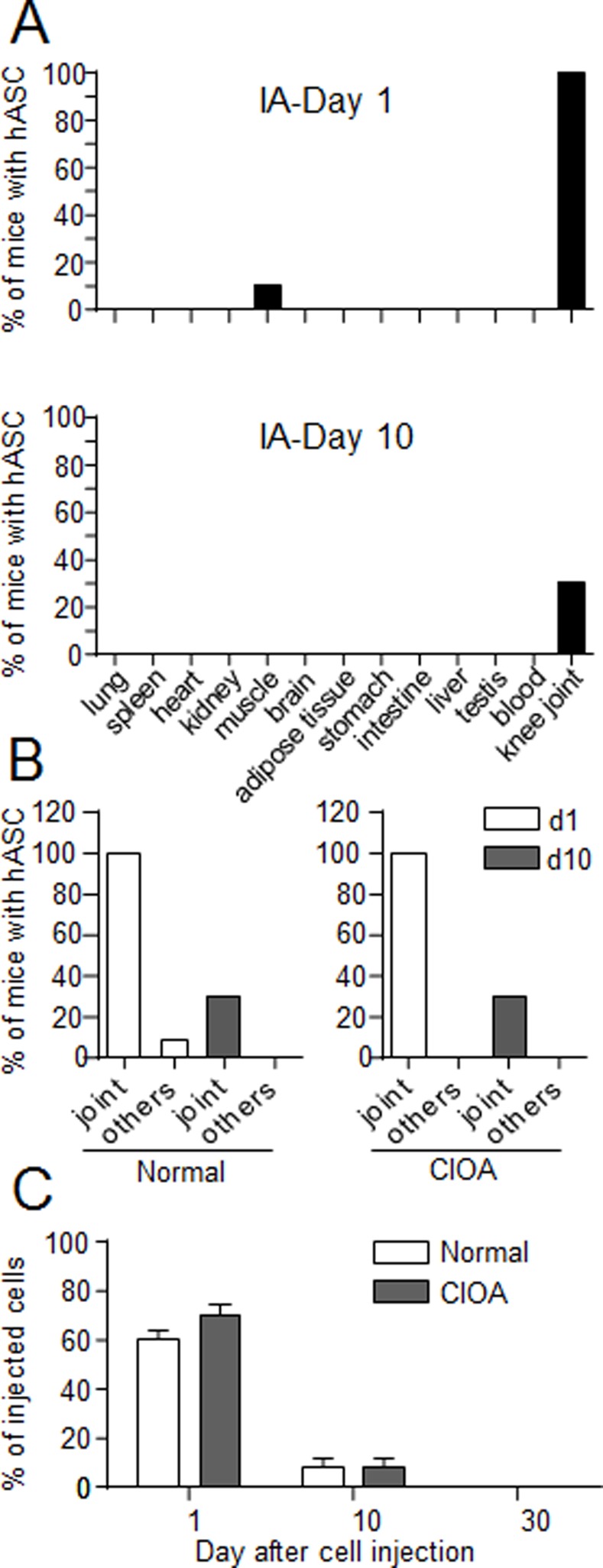
Comparison of the biodistribution of hASCs after IA administration in normal or CIOA mice. (A) Percentage of normal mice in which hASCs were detected in the joints or 12 other organs after IA injection at day 1 or 10. (B) Percentage of normal or CIOA mice in which hASCs were detected in the joints or muscle (others) after IA injection at day 1 or 10. (C) Percentage of hASCs detected in the joint relative to the number of initially injected hASCs in normal or CIOA mice. Results are expressed as the percentage of hASCs as evaluated by *Alu* sequence quantification and normalization using serial dilution of hASCs in murine MSCs ± SEM (standard deviation of the mean).

We investigated whether hASCs may be detected in the joints of CIOA mice at different time points after implantation and whether the persistence and biodistribution of hASCs were altered in the CIOA model as compared to normal mice. We observed the presence of hASCs in the joints of 10/10 and 3/10 of ASC-treated normal mice at day 1 and 10, respectively (Fig. 4A). We also identified hASCs in the *Tibialis anterior* muscle of one mouse at day 1 after cell administration. In CIOA mice, hASCs were detected exclusively in the joints at both time points indicating absence of migration to other organs (Fig. 4B). Interestingly, no differences in the percentage of hASCs present in the joints of normal and CIOA mice were noticed, with respectively 60.5 ± 3.9% and 70.3 ± 4.7% at day 1 and 7.9 ± 4.3% and 8 ± 4.2% in the total group of mice at day 10 (Fig. 3B). At day 30, no cell could be detected in normal or CIOA mice. These data demonstrated that local moderate inflammation does not affect the persistence of cells. They moreover confirm that the therapeutic effect of the cells lasted, also after hASC disappearance.

## Discussion

These data are the first to present the persistence and biodistribution of hASCs after IV or IA injection in arthritic immunocompetent mice, using large scale analysis on 13 different tissues or organs. The important finding of the present study is that inflammation, and notably the degree of inflammation, does not affect the persistence of hASCs as evaluated in two pre-clinical mouse models of arthritis.

Persistence and biodistribution of hASCs are important parameters to evaluate for safety and determination of local interaction and function of cell therapy products. We relied on the quantification of the human specific *Alu* sequences by PCR, which allows high sensitivity of detection of implanted cells without genetic modification or labeling of the cells as previously reported [9]. Although it may be assumed that the number of cells detected in organs is a rough approximation of what is quantified using serial dilution of cultured cells, the approach allowed a long-term follow-up on several organs. In this previous study, we investigated the biodistribution of hASCs in the SCID/Bg immunodeficient mice [9]. In these conditions, we mimicked autologous cell implantation due to the absence of functional B and T lymphocytes but more likely to the absence of NK cells, which are known to efficiently lyse MSCs [18]. A high number of cells had been injected in order to evaluate the safety of clinical grade hASCs, in particular for tumour formation. We demonstrated long-term survival of hASCs (at least 6 months) after IA injection in 60% of the injected mice and noticed the migration of hASCs to some organs, notably muscle, bone marrow and adipose tissue [9]. In the present study, absence of hASC migration to other organs was observed, with the exception of one mouse with cells in the *Tibialis anterior* muscle. Although these differences in migratory profile may be attributed to the immunological status of the mice, they are more likely due to the doses of injected cells, which were 4-fold less than that used in the previous study.

Another difference between these two studies performed in normal mice was the number of cells detected at the same time points. At day 10 in immunocompetent mice, no hASC was observed after IV injection while 8% of hASCs could be detected in the knee joint. By comparison, 1.1% and 12% of hASCs were detected respectively, in lungs and knee joints of immunodeficient mice [9]. At day 30, hASCs were exclusively detected in immunodeficient mice after IA administration. In the joint, hASCs home to the synovial membrane which constitutes a MSC niche [19] and likely a favourable environment for exogenous MSCs. Moreover, the joint space is a restricted environment that mechanically favours cell retention. In accordance, detection of nanoparticle-labelled allogeneic MSCs was also reported up to 7 days following IA injection in another arthritic model [20]. Injection of autologous/syngeneic MSCs allowed persistence for similar or longer periods depending on the OA models used. It fluctuated between 7 days in the mouse model to 2 weeks in rat and 20 days in the rabbit model [12, 21, 22]. Variation in survival time of MSCs in these models may be related to the number of injected cells but also to the sensitivity of the detection method, relying on in vivo luciferase or immunohistochemistry detection. Rapid clearance of hASCs after IV injection was expected by reference to the previous study by Prockop and collaborators who reported a half-life of 24h [23]. Such rapid cell elimination was not observed after IV infusion of allogeneic MSCs, which were completely rejected after a longer period of 40 days [24]. Although the present study did not aim at evaluating cell rejection but rather cell persistence, one could hypothesize that rapid clearance of hASCs is likely to be due to an enhanced host immune response, characterized by detection of interspecies differences in the expression of surface markers and major histocompatibility complex antigens I and II (MHCII). We therefore confirm a longer persistence of hASCs after IA injection and a more rapid disappearance of cells in immunocompetent animals.

Besides comparing the behavior of hASCs in normal mice, the major objective of the study was to investigate whether an inflammatory environment may alter the persistence and distribution of hASCs *in vivo*, with a possible effect on the therapeutic efficacy of the cells. We used the preclinical CIOA and CIA models of the most prevalent rheumatic diseases, OA and RA respectively. Both models are mouse strain—dependent and characterized by inflammation, which is moderate in CIOA and severe in CIA [25]. Efficacy of allogeneic MSCs or xenogeneic hASCs has already been shown in CIA using both preventive and curative approaches, even though some studies report absence of therapeutic benefit likely related to the types of MSCs used [13, 26, 27]. However, efficacy of hASCs was not documented in osteoarthritis models. Using the CIOA model, we showed that hASCs could reduce cartilage degradation of the knee joint and decrease the level of systemic inflammatory mediators demonstrating that hASCs exert a protective effect for at least 35 days. We previously reported that IA administration of syngeneic mMSCs protected against cartilage destruction for a similar period of time [12]. We also showed that mMSCs expressing the GFP marker had disappeared at day 5 after injection. In the present study, hASCs were still detected at day 10. This may be due to the higher number of injected cells (10 fold more) or to the sensitivity of the detection method. In both models, the beneficial effect of MSCs was observed for a long period of time, when cells were no longer detectable. This was recently referred to the hit-and-run mechanism of MSCs where treatment efficacy was not correlated with engraftment duration [28]. However, the question of hASC persistence in a pathological context of inflammation is still poorly answered.

The interesting finding of the present work is that inflammation, which is expected to prime hASCs and up-regulate the expression of immunosuppressive factors, did not affect their survival or biodistribution. First, biodistribution of hASCs was not altered by inflammatory signals. hASCs were retained in the target organs, respective to the route of injection. In the CIA model, a possible migration of hASCs towards the knee joint, which is one of the main inflamed joints, was not observed. One potential explanation may be related to the time of analysis (day 1) or the tissue origin of the cells since after initial concentration in the lungs, bone marrow-derived MSCs have been shown to migrate to injured tissues within 2 to 7 days [23, 29]. However, others report that after 24h, MSC are relocated to other organs while a strong decrease in viability is observed (for review, see [29]). Although we cannot exclude that hASCs had migrated to the joints or other organs at later time points or, that the short survival of cells did not allow the visualization of migratory hASCs at day 10, it is likely that most of the cells rapidly disappeared from lungs. Second and more importantly, survival of hASCs was identical in normal and arthritic mice, as shown by the same number of hASCs detected in the targeted organs, lungs and knee joints in CIA and CIOA respectively. There is now consensus that MSCs are not immune privileged and a number of studies have provided evidence that mismatched MSCs can induce anti-MSC immune responses. They are nevertheless more slowly rejected by the host immune response than other cells and this is dependent on the balance between the expression of immunogenic and immunosuppressive factors by MSCs (for review, see [30]). This implicitly suggests that priming of MSCs by the inflammatory milieu should be required to up-regulate the immunosuppressive factors and enhance their capacity to escape rejection. Accumulating evidence suggests that MSCs can trigger the induction of regulatory T cells or macrophages that may account for their immunoregulatory properties but are short-lived cells *in vivo* [31]. Our results clearly indicate that hASCs were not protected from elimination when present under moderate or severe inflammatory environment. Indeed, while inflammatory signals are required for the immunosuppressive function of MSCs, they do not enhance their capacity to survive *in vivo*.

In conclusion, a number of studies have already investigated the survival and biodistribution of BM-MSCs or ASCs after implantation in different locations, mostly in autologous situations. However, most of the studies did not take into account the pathological environment of the disease itself. In the present study, we report that persistence and biodistribution of hASCs is dependent on the route of administration independently of the immune status of the animals. The important information is that neither inflammation nor the degree of inflammation influences the persistence of hASCs. These findings might have implications in the practical use of MSCs, both in the context of preferred route of administration or state of inflammation in cell activation or rejection.
