# Correction: Survival and Biodistribution of Xenogenic Adipose Mesenchymal Stem Cells Is Not Affected by the Degree of Inflammation in Arthritis

**DOI:** 10.1371/journal.pone.0120406

**Published:** 2015-03-12

**Authors:** The PLOS ONE Staff

There is an error in affiliation 6 for author Marina I. Garin. Affiliation 6 should be: Advanced Therapy Unit, CIEMAT/IIS Fundacion Jimenez Diaz, Madrid, Spain.
